# Development of system using beam's eye view images to measure respiratory motion tracking errors in image‐guided robotic radiosurgery system

**DOI:** 10.1120/jacmp.v16i1.5049

**Published:** 2015-01-08

**Authors:** Mitsuhiro Inoue, Hiroya Shiomi, Hiromitsu Iwata, Junichi Taguchi, Kohei Okawa, Chie Kikuchi, Kosaku Inada, Michio Iwabuchi, Taro Murai, Izumi Koike, Koshi Tatewaki, Seiji Ohta, Tomio Inoue

**Affiliations:** ^1^ Department of Quality Management with Radiotherapy Yokohama CyberKnife Center; ^2^ Department of Radiation Oncology Yokohama CyberKnife Center; ^3^ Department of Neurosurgery Yokohama CyberKnife Center Yokohama Japan; ^4^ Department of Radiology Yokohama City University Graduate School of Medicine Yokohama Japan; ^5^ Department of Radiation Oncology Osaka University Graduate School of Medicine Osaka Japan; ^6^ Department of Radiation Oncology Nagoya Proton Therapy Center Nagoya City West Medical Center Nagoya Japan; ^7^ Department of Radiology Nagoya City University Graduate School of Medical Science Nagoya Japan

**Keywords:** lung, CyberKnife, Synchrony system, SBRT

## Abstract

The accuracy of the CyberKnife Synchrony Respiratory Tracking System (SRTS) is considered to be patient‐dependent because the SRTS relies on an individual correlation between the internal tumor position (ITP) and the external marker position (EMP), as well as a prediction method to compensate for the delay incurred to adjust the position of the linear accelerator (linac). We aimed to develop a system for obtaining pretreatment statistical measurements of the SRTS tracking error by using beam's eye view (BEV) images, to enable the prediction of the patient‐specific accuracy. The respiratory motion data for the ITP and the EMP were derived from cine MR images obtained from 23 patients. The dynamic motion phantom was used to reproduce both the ITP and EMP motions. The CyberKnife was subsequently operated with the SRTS, with a CCD camera mounted on the head of the linac. BEV images from the CCD camera were recorded during the tracking of a ball target by the linac. The tracking error was measured at 15 Hz using in‐house software. To assess the precision of the position detection using an MR image, the positions of test tubes (determined from MR images) were compared with their actual positions. To assess the precision of the position detection of the ball, ball positions measured from BEV images were compared with values measured using a Vernier caliper. The SRTS accuracy was evaluated by determining the tracking error that could be identified with a probability of more than 95% (Ep95). The detection precision of the tumor position (determined from cine MR images) was <0.2 mm. The detection precision of the tracking error when using the BEV images was <0.2 mm. These two detection precisions were derived from our measurement system and were not obtained from the SRTS. The median of Ep95 was found to be 1.5 (range, 1.0–3.5) mm. The difference between the minimum and maximum Ep95 was 2.5 mm, indicating that this provides a better means of evaluating patient‐specific SRTS accuracy. A suitable margin, based on the predicted patient‐specific SRTS accuracy, can be added to the clinical target volume.

PACS number: 87.53.Ly

## I. INTRODUCTION

The CyberKnife Robotic Radiosurgery System (Accuray, Inc., Sunnyvale, CA) is a frameless image‐guided stereotactic radiotherapy system. A 6 MV linac is mounted on a high‐precision, six‐axis robotic manipulator arm. Beams are accurately delivered to the target through the collimator. Originally, the CyberKnife was used to treat brain and head/neck tumors.^1^, ^2^ Technological developments then allowed the system to be applied to extracranial regions with no respiratory displacement, such as in the treatment of spinal cord tumors.^3^, ^4^ Subsequent advances have enabled the treatment of extracranial regions subject to movement as a result of respiratory displacement, such as in the case of the treatment of lung tumors.^5^, ^6^

The SRTS is a CyberKnife subsystem, developed to irradiate extracranial tumors that move as a result of respiration. It continuously synchronizes the beam delivery with the moving tumors. With the SRTS, patients can breathe normally during treatment, and the treatment margins are reduced through precise tracking.^7^, ^8^, ^9^

The SRTS relies on the correlation between the ITP and the EMP. Before the start of each treatment fraction, the ITP is measured by using orthogonal X‐ray images at no fewer than eight data points. The external chest/abdominal marker is continuously monitored by using optical LEDs. The correlation model is a linear or polynomial fit between the 3D ITP and the simultaneous EMP, and is automatically established by the system. It is updated by acquiring additional X‐ray images during treatment. At our institution, this is typically done every 45 to 60 sec. The SRTS estimates the ITP from the EMP by applying a correlation model, and then delivers the beams to the moving tumor. However, the necessary data processing, communication with the robotic controller, and the inertia of the robotic manipulator and linear accelerator all contribute to the delay that is incurred when determining the position for the beam. Therefore, a prediction method is employed to compensate for this delay.^9^, ^10^, ^11^

The accuracy of the CyberKnife was first evaluated in 1996,^(12)^ and several subsequent studies of its accuracy have been published.^13^, ^14^, ^15^, ^16^, ^17^ The overall system error was defined as the distance between the centroids of the planned and delivered dose distributions. The first reported study of the accuracy of the SRTS was performed by using a similar method in 2004.^(9)^ A method that relied on analyzing the shift in the centroid of the 70% isodose line in a sphere with delivered dose distributions was accurate enough to evaluate the dose delivery for nonmoving regions; however, it was not sensitive enough for application to moving regions. Therefore, several different methods were applied to these investigations, such as comparing the isodose distribution and the gamma map between the planned and delivered dose distributions, and for the subsequent retrospective analysis of clinical data.^7^, ^10^, ^11^, ^18^, ^19^, ^20^, ^21^

Any poor correlation between the ITP and EMP and/or an irregular breathing pattern could have an adverse effect on the precision of the dose delivery because the SRTS utilizes both a correlation model and the prediction method. Therefore, it is possible that the SRTS accuracy is patient‐dependent, meaning that the treatment margin should be changed for each individual. Most previous reports evaluated the SRTS accuracy retrospectively and no studies attempted to estimate the pretreatment SRTS accuracy.^7^, ^10^, ^11^, ^20^

The purpose of this study was to develop a system for performing pretreatment statistical measurement of the SRTS's tracking error and so enable the patient‐specific prediction of the SRTS accuracy.

## II. MATERIALS AND METHODS

### A. Patient characteristics

We analyzed 23 lung treatment patients for whom it was possible to obtain respiratory motion data from cine magnetic resonance (MR) images and who had been treated with the current version (3.1.1) of the SRTS between September 2013 and July 2014. The median age of the 23 patients was 74 yrs (ranging from 53 to 86 yrs, and consisting of 14 males and 9 females). The tumors were located in the right lower lobe of the lung in ten patients, in the left lower lobe in six patients, in the right upper lobe in three patients, in the left upper lobe in three patients, and in the right middle lobe in one patient. All of the study participants provided informed consent, and the design of the study was approved by our institutional review board. We used not only respiratory motion data from the patients receiving treatment, but also sinusoidal wave data as a basic case for this study.

### B. Dynamic motion phantom

We used the CIRS Dynamic Thorax Phantom Model 008A (Computerized Imaging Reference Systems, Inc., Norfolk, VA) as a dynamic motion phantom (DMP). This DMP consists of two separate platforms, namely a target motion simulator and an external skin motion simulator; these are independently controlled by CIRS motion‐control software. The DMP generates three‐dimensional target motion and has a manufacturer‐stated absolute position accuracy of 0.1 mm. Motion range was up to 50 mm, 10 mm, and 10 mm in the craniocaudal (C–C), anterior–posterior (A–P), and left–right (L–R) directions, respectively. The DMP applied different waveforms to C–C, A–P, and L–R. In addition, the amplitudes and phase shifts for the C–C, A–P, and L–R directions were adjusted independently.

**Figure 1:**
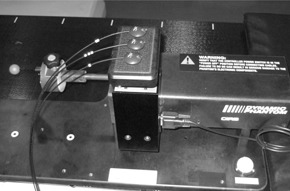
Dynamic motion phantom (DMP): a 20 mm diameter plastic ball, with a gold marker at its center, was used as a target. The ball was set on the platform for target motion simulation and three optical markers were set up on the platform for external skin motion simulation.

A 20 mm diameter plastic ball, with a gold marker at its center, was used as the target. The ball was set up on a platform to simulate the target motion, and three optical markers were set up on the platform to simulate the external skin motion (Fig. 1). The DMP was actuated based on a dataset for a sinusoidal wave as a simple case, and the respiratory motion data for the 23 patients as a clinical case. The sinusoidal wave data and respiratory motion data are described in detail below.

### C. Sinusoidal wave data and respiratory motion data

Sinusoidal wave data: the range of motion was 20 mm and 10 mm for the superior–inferior (S–I) direction (with the target motion simulator) and A–P direction (with the external skin motion simulator), respectively; the motion frequency was 16 cycles/min.

Respiratory motion data: an MR scan was performed with a 1.5‐Tesla whole‐body clinical MR scanner (Magnetom Symphony Syngo; Siemens Medical Solutions, Munich, Germany) using a CP body array flex coil and a spinal coil for the lung tumor patients. The sagittal plane was obtained by true fast imaging with a steady‐state precession sequence. The sequence parameters were as follows: repetition time/echo time: 4.3 ms/1.73 ms; field of view: (228 to 333)×500 mm; flip angle: 20°; bandwidth: 449 Hz; image matrix: (176 to 256)×384; image acquisition time: 0.2 to 0.3 sec; slice thickness: 8 mm. The sagittal plane was set to that plane which passes through the center of the tumor. We acquired 512 continuous images. This scan was repeated three times, and then the three sets of images were merged.

A test tube containing a contrast agent was placed on the surface of the patient's abdomen as an external marker. The position was almost the same as that of the external marker for the SRTS. The diameter of the test tube was 14 mm. All of the sagittal plane images included both the tumor and the test tube.

Respiratory motion data for the ITP and EMP were collected from the cine MR images using BreathingData (software developed in‐house by author M. I. for this system) that implements template matching based on a zero mean normalized cross‐correlation function. Template matching was implemented by first picking out parts of the tumor and external marker to use as a template. The software then searched for similar regions on the other images, by comparing them with the tumor template and external marker template; this was done for every image. BreathingData determined the tumor and external marker positions by calculating the position of the centroid of some of the pixels constituting the tumor and external marker. The sagittal plane yielded the simultaneous motion of the lung tumor in the C–C and A–P directions and the motion of the surface of the chest/abdomen in the A–P direction.

**Figure 2:**
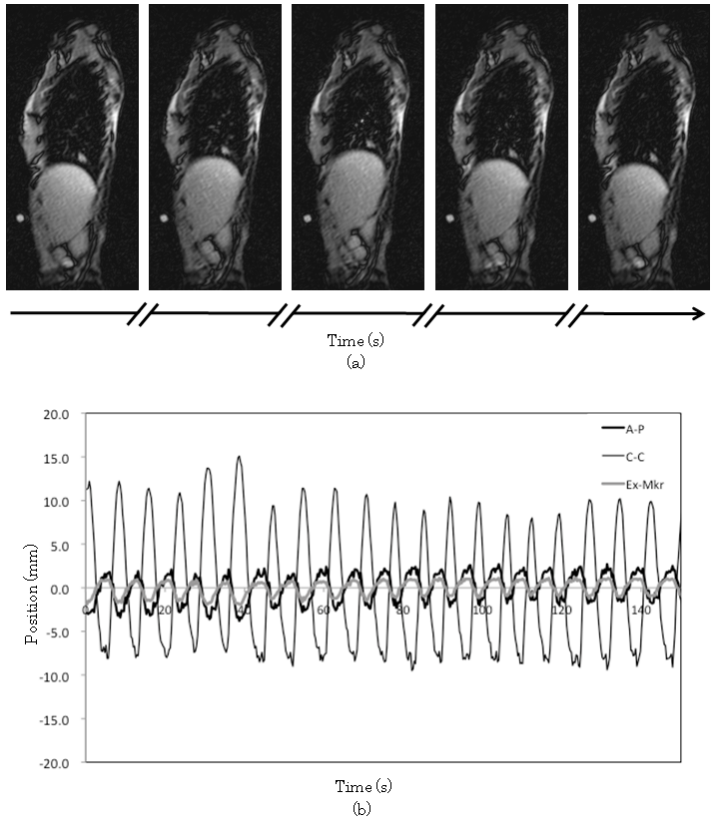
Sample sagittal cine MRI (a) and sample respiratory motion of tumor (b) in antero–posterior (A–P) and craniocaudal (C–C) directions and that of extra marker (Ex‐Mkr) in A–P direction. Sagittal images were acquired every 0.2 to 0.3 sec. Respiratory motion data in 0.02 to 0.03 sec intervals were calculated using cubic spline interpolation.

To reduce noise, smoothing was applied to all of the respiratory motion data by using a simple moving average, which was the unweighted mean of the two points. Then, respiratory motion data were calculated for 0.033 sec intervals by using cubic spline interpolation for the dynamic motion phantom. Respiratory motion data were obtained from continuous imaging of three sets for about 330 sec. Figure 2 shows sample cine MR images with the sagittal plane and respiratory motion data from the sagittal plane.

### D. Measurements

Treatment plans were created based on static CT scans and standard CyberKnife treatment parameters. Each plan utilized ten beams, with several different source positions. All of the beams in this plan aimed at the center of the ball. All of the beams were set to 200 MU for 15 sec of data acquisition.

**Figure 3:**
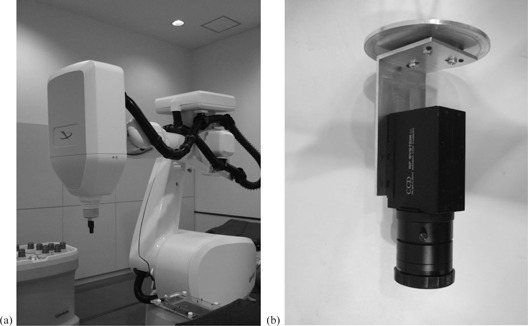
CCD camera (a) mounted on the linear accelerator (linac) head. Beam's eye view images from the CCD camera were recorded during ball tracking by the linac. CCD camera and custom‐built jig (b). The central axis of the CCD camera was aligned with that of the linac beam by using the custom‐built jig.

The DMP motion used sinusoidal wave data for a simple case and the patients’ respiratory motion data for a clinical case. Then, the CyberKnife was moved under the control of Synchrony in demonstration mode, with a CCD camera (PC‐5VM; RF System Lab, Nagano, Japan.) mounted on the head of the linac (Fig. 3(a)). A custom‐built jig was attached to a CCD camera (Fig. 3(b)). The jig was designed to accurately fit a 10 mm collimator and adjusted to align the center of the BEV image with the central axis of the linac beam. Before the start of measurement, the ITP was measured at 12 to 15 data points by using orthogonal X‐ray imaging, until over 90% of the respiratory cycle was covered. It was not updated by acquiring additional X‐ray images during the measurement.

Beam's eye view (BEV) images from the CCD camera were recorded as the linac tracked the ball. The central axis of the CCD camera (center of BEV image) was matched to the central axis of the linac beam by using a custom‐built jig. The ball was stationary at the center of the BEV image when tracking was complete, but would be shifted from the center of the BEV image when the tracking was not complete. The tracking error was defined as the distance from the center of the BEV image to the center of the ball in the BEV images. The tracking error was measured at 15 Hz using VideoCapture (software developed in‐house by author H. S. for analyzing the tracking error). The software used a 320×240 pixel image with a resolution of about 0.5 mm at the distance of the ball.

### E. Precision of position detection using MR image

A test tube containing a contrast agent and an acrylic cube phantom filled with water were placed on the MR couch. The test tube was too small to produce an MR signal, so we used the acrylic cube phantom filled with water. The test tube was rotated relative to the A–P axis as the test tube position was shifted 0.5 mm in the C–C direction in sagittal plane, which was moved 5 mm in L–R direction. Sagittal images were taken for 13 different planes at 5 mm intervals. The MR sequence was used to obtain respiratory motion data for each patient. The test tube position was determined from MR images using BreathingData and was compared with the nominal test tube position.

### F. Precision of ball position detection when using VideoCapture

The ball was placed on the target motion simulator of the DMP and was moved within a range of −7 mm to +7 mm in increments of 0.5 mm. A Vernier caliper was attached to the target simulator to obtain the true value of the movement of the ball. A CCD camera was mounted on the linac head, placed perpendicular to the axis of movement of the ball. The orientation of the CCD camera was adjusted to be parallel and perpendicular to the axis of movement of the ball on the BEV image. The ball positions were determined from the BEV images using VideoCapture and were compared with the values measured by the Vernier caliper.

### G. Accuracy of SRTS

We calculated the tracking error that could be tracked with a probability in excess of 95% (Ep95) for each beam direction. The SRTS accuracy was defined as the median value of Ep95 for ten beams (Ep95med).

## III. RESULTS

**Figure 4:**
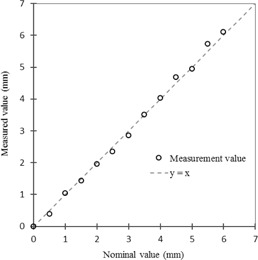
Relationship between values measured by BreathingData based on MR images (y‐axis) and nominal test tube position (x‐axis).

Figure 4 shows the relationship between the values obtained with BreathingData from MR images and the nominal test tube position. The mean value and standard deviation of the position difference was 0.00±0.13 mm. The difference for every position was less than 0.2 mm.

**Figure 5:**
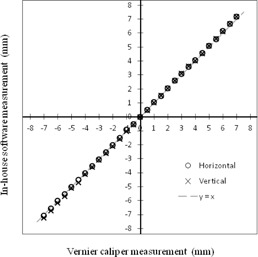
Relationship between values measured with a Vernier caliper (x‐axis) and those measured by VideoCapture based on BEV images of the ball position (y‐axis).

Figure 5 shows the relationship between the values measured with a Vernier caliper and those measured by VideoCapture using BEV images of the ball position. The mean value and standard deviation of the position differences were 0.05±0.07 mm and 0.01±0.12 mm in the horizontal and vertical directions, respectively. The minimum and maximum values of the position differences were −0.05 mm to 0.21 mm and −0.19 mm to 0.21 mm in the horizontal and vertical directions, respectively. The maximum absolute difference for all positions was 0.21 mm. However, the difference was less than 0.2 mm within a range of −5 mm to 5 mm.

**Figure 6:**
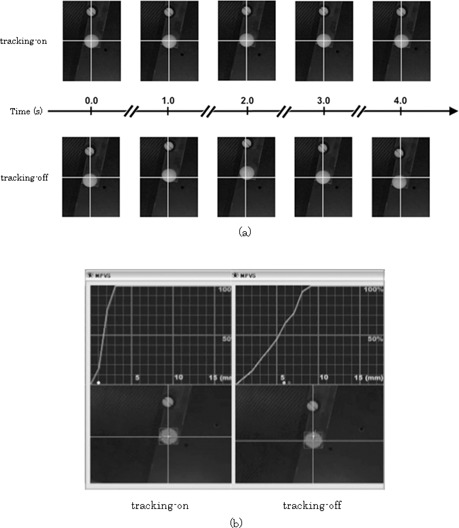
Beam's eye view images from CCD camera (a): top panel images correspond to the “tracking‐on” setting, and those in the bottom panel were obtained with the “tracking‐off” setting. In the top panel, the ball was placed approximately at the center of the BEV images, whereas in the bottom panel images it was placed far from the center of the BEV images due to its motion. Screen of VideoCapture (b): “tracking‐on” setting (left) and “tracking‐off” setting (right). The top panels show the tracking error probability histograms.

Representative BEV images from the CCD camera are shown in Fig. 6(a). The images in the top panel were taken with the “tracking‐on” setting, while those in the bottom panel were taken with the “tracking‐off” setting, for reference. In the “tracking‐on” panel, the ball was positioned approximately at the center of the BEV image, whereas in the “tracking‐off” panel it was far from the center of the image due to its motion. Fig. 6(b) shows the screen of VideoCapture; the left side corresponds to the “tracking‐on” setting, and the right side to the “tracking‐off” setting. The upper panels show the tracking error probability histograms.

**Figure 7:**
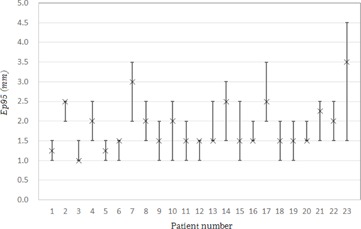
Minimum, maximum, and median values of Ep95 for each patient.

**Figure 8:**
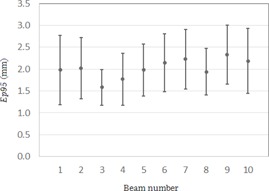
Average and standard deviation values of Ep95 for each beam.

**Figure 9:**
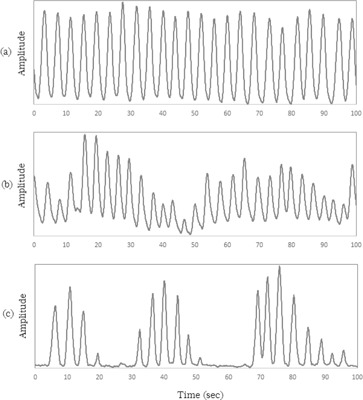
Respiratory motion data for Patients 13 (a), 18 (b), and 23 (c).

Ep95med for the sinusoidal wave data was 1.0 mm. Table 1 lists the tumor location, the mean value of the range of motion of the tumor motion in the A–P and C–C directions, the motion frequency, and Ep95med for all cases. The mean values of the range of motion of the tumor in all cases ranged from 0.5 mm to 6.8 mm (median=3.9 mm) for the A–P direction, and from 3.0 mm to 29.0 mm (median=9.4 mm) for the C–C direction. The median of the motion frequency was 15 cycles per min (range 7 to 35). Figure 7 shows the minimum, maximum, and median values of Ep95 for each patient. The median value of Ep95med for all of the patients was 1.5 mm (the range was 1.0 to 3.5). Figure 8 shows the average and standard deviation of Ep95 for each beam. The average value of Ep95 for all the beams was 2.0 mm (the range was 1.6 to 2.3). The average value of Ep95 for Beam 4 was 1.6 mm, which was 0.4 mm less than the average for all the beams. Figure 9 shows the characteristic respiratory motion data for Patient 13, Patient 18, and Patient 23.

**Table 1:** Location of tumor, range of motion of tumor, motion frequency, and Ep95med

		*Range of Motion (mm)*			
*Case*	*Location*	*A–P*	*C–C*	*Frequency (cycles/min)*	*Ep95med (mm)*
1	L upper lobe	5.4±1.3	9.4±3.0	23	1.3
2	L lower lobe	5.2±0.6	14.6±3.3	21	2.5
3	L lower lobe	0.5±0.4	6.4±2.1	22	1.0
4	R lower lobe	1.8±0.8	15.8±2.0	16	2.0
5	R lower lobe	2.4±0.7	5.9±0.7	20	1.3
6	L lower lobe	1.5±0.8	11.2±1.2	16	1.5
7	R upper lobe	6.8±0.9	29.0±3.8	13	3.0
8	L lower lobe	2.0±0.4	24.9±2.7	12	2.0
9	R upper lobe	4.1±0.5	4.5±0.6	19	1.5
10	R middle lobe	4.0±0.5	8.2±0.7	7	2.0
11	R lower lobe	2.8±1.1	7.2±3.3	15	1.5
12	L upper lobe	4.7±0.8	6.3±0.8	15	1.5
13	R lower lobe	2.0±0.6	10.0±0.5	15	1.5
14	R lower lobe	4.3±0.6	10.4±1.5	19	2.5
15	R lower lobe	4.5±1.1	3.0±0.7	20	1.5
16	R lower lobe	4.8±2.0	13.3±3.7	14	1.5
17	R lower lobe	1.1±0.6	17.4±2.9	18	2.5
18	L upper lobe	2.9±1.3	5.9±2.4	15	1.5
19	R lower lobe	1.1±0.2	9.0±1.1	35	1.5
20	R upper lobe	5.9±2.1	6.4±1.3	13	1.5
21	L lower lobe	3.9±2.1	14.7±4.4	15	2.3
22	L lower lobe	3.9±0.6	10.8±0.1	15	2.0
23	R lower lobe	0.7±0.6	6.0±6.5	10	3.5

L=left; R=right; A−P=anterior‐posterior; C−C=craniocaudal; Ep95med=median value of the tracking error value that could be tracked with a probability higher than 95% for ten beams

## IV. DISCUSSION

The position difference between the values measured from MR images using BreathingData and the nominal test tube position was less than 0.2 mm in all positions. The difference between the values measured for the ball position with a Vernier caliper and those measured from BEV images using VideoCapture was less than 0.2 mm over a range of −5 mm to 5 mm. Therefore, the precision of our method was determined as being acceptable for statistical measurement of the SRTS's tracking error.

One of the limitations of this study was that we could not evaluate the tracking error in the direction of the central beam axis because the target motion was captured by a 2D BEV image. The average value of Ep95 for Beam 4 was the smallest; the angle of Beam 4 and the C–C direction of DMP were smaller than those of the other beams. This result indicates that the target motion captured by a 2D BEV image would change depending on the direction of the target motion and/or the irradiation angle. In other words, the dosimetric consequences may be overestimated as a result of a very slight movement of the target when the tracking depends on the irradiation angle but the tracking error is assessed only from the log files. Although a tracking error in the vertical direction could affect the dose delivery, a tracking error in the direction of the central beam axis could have no adverse clinical impact. Because the CyberKnife's beam profile is not flat, a steep falloff of the penumbra could shift the dose by >10% even for a displacement of as little as 1 mm. A displacement of 1 mm in the direction of the central beam axis could lead to a difference in the dose of <1%. Therefore, the use of a 2D BEV image is acceptable for clinical use.

Previous reports suggested that the respiratory external marker motion does not always accurately correspond to the 3D internal target motion and that this may affect the SRTS accuracy.^7^, ^10^, ^22^, ^23^ Therefore, it is important to consider the correlation between the motions and the regularity of the breathing pattern when evaluating the patient‐specific SRTS accuracy. Our method takes tracking errors arising from such factors into account. In this study, we used cine MR images to acquire respiratory motion data for the ITP and EMP. Using BEV images, the DMP can reproduce both the ITP and EMP motion and the measured SRTS's tracking error. This study was not able to account for tumor motion in the L–R direction, however, because we used the sagittal plane to simultaneously obtain the motion of the tumor and the body surface. Seppenwoodle et al.^(24)^ showed the 3D tumor motion, and indicated that the L–R direction had the smallest amplitude of the overall tumor motion. Be that as it may, we believe that this limitation does not present a significant problem for any clinical case.

The Ep95med in our study was 1.5 mm, larger than that obtained with a similar phantom in a previous report.^(20)^ Because that study used the same data for the ITP and EMP tests, the result did not incorporate the tracking error determined from the correlation between the ITP and EMP. Our study of clinical cases used independent data that included device‐ and patient‐specific tracking error factors. The results reported by Wong et al.^(20)^ were similar to those for Ep95med for the sinusoidal wave data, which did not include any tracking errors from the ITP–EMP correlation. Therefore, we assumed that that independent data for ITP and EMP were necessary to evaluate the SRTS tracking error for a clinical case.

Although the respiratory motion data for Patient 18 exhibited an irregular breathing pattern, the value of Ep95med was 1.5 mm. Wong et al.^(20)^ also stated that SRTS could track a tumor with a high degree of accuracy despite irregular breathing. However, the value of Ep95med for Patient 23 was 3.5 mm, which was the maximum value of all the patients. The respiratory motion data for Patient 23 exhibited an irregular breathing pattern and had a breath‐holding time exceeding 10 sec due to apnea. It might be inferred from the Patient 23 data that the prediction model does not function well with an irregular breathing pattern like that of Patient 23.

The difference between the minimum and maximum value of the Ep95med was 2.5 mm. This difference indicates that the SRTS accuracy is patient‐dependent. Therefore, we concluded that it was better to measure the SRTS tracking error before any treatment, to enable the prediction of the patient‐specific accuracy. Furthermore, it was better to add a suitable margin for each patient. As such, our institution adds the value of Ep95med to each patient as a margin for the tracking error.

## V. CONCLUSIONS

The SRTS's tracking error can be statistically measured prior to treatment. A suitable margin can then be added according to the predicted patient‐specific SRTS accuracy. The application of a suitable margin to the clinical target volume will prevent surrounding structures from being affected.

## Supporting information

Supplementary Material
