# Supplementary figures and images for: Development of system using beam's eye view images to measure respiratory motion tracking errors in image‐guided robotic radiosurgery system
Source: J Appl Clin Med Phys. 2015 Jan 8;16(1):100–11. doi: 10.1120/jacmp.v16i1.5049 (PMC5689998; doi:10.1120/jacmp.v16i1.5049)

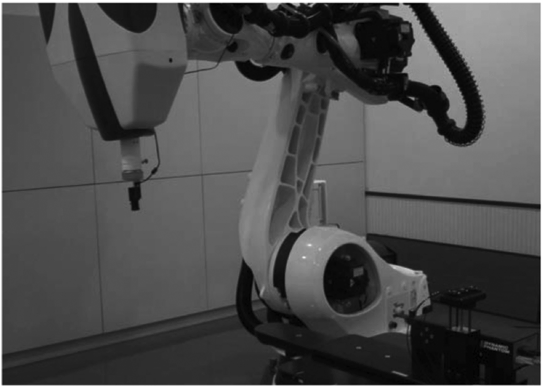

Supplement: Supplementary file 1 — Supplementary Material [file ACM2-16-100-s001.png]
